# Immunological Cross-Reactivity between Malaria Vaccine Target Antigen P48/45 in *Plasmodium vivax* and *P*. *falciparum* and Cross–Boosting of Immune Responses

**DOI:** 10.1371/journal.pone.0158212

**Published:** 2016-07-20

**Authors:** Yi Cao, Geetha P. Bansal, Kristen Merino, Nirbhay Kumar

**Affiliations:** Department of Tropical Medicine, School of Public Health and Tropical Medicine, and Vector-Borne Infectious Disease Research Center, Tulane University, New Orleans, Louisiana, 70112, United States of America; Ehime University, JAPAN

## Abstract

In general, malaria immunity has been suggested to be species specific with very little, if any, known cross-reactivity between *Plasmodium vivax* and *P*. *falciparum*, both of which are responsible for >90% of human malaria, and co-endemic in many countries. It is therefore believed that species-specific immunity may be needed to target different species of *Plasmodium*. Pfs48/45 and Pvs48/45 are well established targets in the sexual stages of the malaria parasites, and are being pursued for the development of transmission blocking vaccines. Comparison of their sequences reveals 61% and 55% identity at the DNA and protein level, respectively raising the possibility that these two target antigens might share cross-reacting epitopes. Having succeeded in expressing recombinant Pfs48/45 and Pvs48/45 proteins, we hypothesized that these proteins will not only exhibit immunological cross–reactivity but also cross-boost immune responses. Mice were immunized with purified recombinant proteins using CFA, Montanide ISA-51 and alum as adjuvants, and the sera were analyzed by ELISA, Western blotting and indirect fixed and live IFA to address the hypothesis. Our studies revealed that Pvs48/45-immune sera showed strong cross-reactivity to full length Pfs48/45 protein, and the majority of this cross reactivity was in the amino-terminal and carboxyl-terminal sub-fragments of Pfs48/45. In cross-boosting experiments Pfs48/45 and Pvs48/45 antigens were able to cross-boost each other in mouse immunization studies. Additionally we also noticed an effect of adjuvants in the overall magnitude of observed cross-reactivity. These studies may have significant implications for immunity targeting transmission of both the species of malaria parasites.

## Introduction

The life cycle of the malaria parasite is complex, consisting of three developmental stages: in the liver and red blood cells of vertebrate hosts and in *Anopheles* mosquitoes, serving as transmission vectors. Hence, the malaria antigens identified as vaccine candidates can be broadly grouped as pre-erythrocytic stage, blood stage and transmission blocking antigens. Antigens in the sexual stages of malaria parasites represent targets of malaria transmission blocking vaccines (TBV), which elicit immunity in the vertebrate host and block the parasite sexual development in the mosquito vector [1]. Current TBV development efforts are focused on antigens expressed on the surface of gametocytes developing as intra-erythrocytic sexual stages in the vertebrate blood, such as Pfs48/45 [2] and Pfs230 [3] in *P*. *falciparum* and Pvs48/45 [4] in *P*. *vivax*, and on the surface of the gamete, zygote and ookinete developing in the mosquito, such as Pfs25 and Pfs28 [5] in *P*. *falciparum* and Pvs25 and Pvs28 [6] in *P*. *vivax*. The P48/45 proteins are conserved in *Plasmodium* spp. and belong to the six-cysteine protein family, which also includes other members such as Pfs230, Pfs47, P36, P52 and Pf12 [3, 7–10]. The presence of multiple conserved disulfide bonds in P48/45 proteins ensures proper conformational folding which is critical for protein function, and transmission blocking antibody responses are directed against such conformational epitopes. The P48/45 antigen is one of the leading TBV candidates based on the following facts: gene disruption in *P*. *falciparum* and *P*. *berghei* (a rodent malaria parasite) resulted in infertile male gametes [11], the recombinant Pfs48/45 elicited antibodies in experimental animals that showed potent transmission blocking activity in membrane feeding assay (MFA) [12–15], immunization with DNA vaccines encoding Pvs48/45 [16] or Pfs48/45 [Datta et al, submitted] elicited specific antibodies in mice that reduced oocyst number in the mosquito midgut in MFA, and antibodies against Pfs48/45 in human sera during natural infection revealed a strong correlation with transmission reducing activity in endemic area [17, 18].

A few studies have probed the immune cross-reactivity of antigens in pre-erythrocytic and erythrocytic asexual stages of different *Plasmodium* species. For example, a *P*. *vivax* circumsporozoite protein (CSP)-based vaccine (VMP001) containing conserved CSP regions generated antibodies that could also recognize the CSP on the surface of *P*. *falciparum* and *P*. *berghei*. The same study also revealed that the anti-serum reduced the heterologous *P*. *berghei* infection despite the low level of cross-reactive antibody titers [19]. The cell-traversal protein expressed in ookinetes and sporozoites (CelTOS) is another example of a highly conserved molecule across *Plasmodium* species. The recombinant PfCelTOS elicited antibodies in mice that showed cross-reactivity to the heterologous *P*. *berghei* sporozoites and induced cross-species protection against *P*. *berghei* challenge [20]. Studies using radiation attenuated sporozoites or genetically attenuated sporozoites have also shown protective efficacy against challenge infection with not only homologous but also heterologous *Plasmodium* species [21].

Among the erythrocytic asexual blood-stage antigens, immune sera and monoclonal antibodies against apical membrane antigen 1 (AMA-1) showed only limited cross reactivity between *P*. *falciparum* and *P*. *vivax* [22]. *P*. *falciparum* CLAG9 (cytoadherence-linked asexual gene) peptides elicited antibodies in mice that recognized the infected red blood cells from both *P*. *falciparum* and *P*. *vivax* and the sera from *P*. *vivax* patients also reacted strongly with the PfCLAG9 peptides [23]. Similar studies using sera from people infected with *P*. *falciparum* and *P*. *vixax* have revealed cross reactivity of merozoite surface protein 5 (MSP5)-specific antibodies [24]. Further support for cross-species reactivity and immune protection has been provided by studies on immunization with murine malaria parasites [25]. While evidence for cross-species immune reactivity has been reported for antigens in the pre-erythrocytic and erythrocytic asexual stages, no such cross-reactivity has been reported for any of the sexual stage antigens. Pfs48/45 and Pvs48/45 share 61% identity at the level of DNA sequence and 55% identity at the level of protein sequence (S1 Fig). The availability of *E*. *coli*–expressed recombinant Pfs48/45 and Pvs48/45 offered us the opportunity to investigate the question of immune cross reactivity and cross-boosting of immune responses. While such cross reactivity may be directed against conformational and non-conformational epitopes, demonstration of any immunological cross-reactivity and cross-boosting may have significant impact on future development of effective transmission blocking immunogens targeting both the species of *Plasmodium*.

The majority of malaria morbidity and mortality in the world is attributed to *P*. *falciparum*, while *P*. *vivax* causes infection in broader endemic geographic areas and accounts for the majority of malaria cases outside of Africa. In many endemic regions, the patients are frequently co-infected with both of them [26, 27]. While species-specific vaccine targeting either of these species is expected to effectively reduce transmission, cross-reactive epitopes in leading vaccine antigens may induce immune responses that might be cross-protective against multiple human malaria parasites. Further significance of such immune cross-reactivity may even imply cross-boosting of immune responses, especially between *P*. *falciparum* and *P*. *vivax*.

## Materials and Methods

### Expression and purification of Pfs48/45 and Pvs48/45

The cloning, expression, purification and characterization of recombinant full-length Pfs48/45 (Genbank accession number AF356146) (minus N-terminal signal and C-terminal anchor sequences) was as described [13]. A similar protocol was used to obtain recombinant full-length Pvs48/45 (PlasmoDB accession number PVX_083235) with minor modifications. Briefly, the codon harmonized sequence of Pvs48/45 was synthesized (Genscript), and cloned into the expression vector pET(K-) between NdeI and NotI restriction sites. An extra adenosine was inserted between the NotI and Xhol in pET(K-) to make the C-terminal 6X histidine tag in the open reading frame of recombinant Pvs48/45. After IPTG induction, the bacterial pellet was lysed by microfluidization in PBS (pH 8.0), and sequentially extracted with PBS containing 1%Tween-80 (1 hour at 4°C) and PBS containing 1.5% L-lauroylsarcosine sodium salt (sarcosyl) at room temperature for 1 hour. The supernatant was diluted 5 times to lower sarcosyl concentration to 0.3%, and then passed through Ni^2+^-NTA column (QIAGEN), followed by elution of protein with 350 mM imidazole and 300 mM NaCl in PBS (pH 8.0). Fractions containing eluted protein were pooled and dialyzed using PBS (pH 8.0) containing 350mM NaCl at 4°C. The quality of protein was assessed by SDS-PAGE under non-reducing and reducing conditions and Western blotting using anti-(His)6 monoclonal antibody (Clontech). Protein concentration was estimated using BCA Protein Assay kit (Pierce) and the endotoxin level in the protein was measured with LAL chromogenic endotoxin quantification kit (Pierce).

### Expression of sub-fragments of Pfs48/45

To express recombinant fragments of Pfs48/45, the full length Pfs48/45 sequence without signal and anchor sequences was divided into five overlapping sub-fragments (amino acid residues: F1, 28–127; F2, 108–207; F3, 188–287; F4, 268–367 and F5, 348–427). Coding sequences for all the fragments were amplified by PCR from the codon harmonized sequence of Pfs48/45, cloned into pRSET-A vector (Invitrogen) and expressed in *E*. *coli* BL21 (DE3) competent cells. After induction with 1.0 mM IPTG, the cell pellets were extracted with 2% sarcosyl and fragments were purified using Ni^2+^-NTA agarose beads. The bound proteins were eluted with 400 mM imidazole, 500mM NaCl, 0.3% sarcosyl and 10% glycerol in PBS (pH 7.4). Fractions containing expressed protein fragments were pooled and then dialyzed against PBS (pH 7.4) containing 350mM NaCl and 10% glycerol and stored at -80°C.

### Mouse immunizations

Three groups (N = 5 per group) of female BALB/c mice were immunized with 0.01 mg of recombinant Pvs48/45 formulated respectively in complete Freund's adjuvant (CFA) (Sigma), Montanide ISA-51 (Seppic) and aluminum hydroxide (alhydrogel) (Brenntag Biosector) through the intraperitoneal (i.p.) route in a total volume of 0.1 mL/mouse. Mice received two booster doses at 3-week intervals with the same quantity of protein in respective adjuvants except that incomplete Freund's adjuvant (IFA) was used for mice immunized with CFA. Blood was collected on day 0 and 2 weeks after primary immunization and each boost. Similarly, female BALB/c (N = 5 per group) were immunized with 0.01 mg of recombinant Pfs48/45 formulated in CFA, Montanide ISA-51 and aluminum hydroxide through the i.p. route, boosted and bled as above.

For the cross-boosting studies, two groups of BABL/c mice (N = 5 per group) were primed with 0.01 mg Pfs48/45 and Pvs48/45 in CFA, respectively, and boosted 3 weeks later with 0.01 mg of the heterologous proteins in IFA (Pfs48/45 prime—Pvs48/45 boost or Pvs48/45 prime—Pfs48/45 boost). In parallel, another two groups of BABL/c mice (N = 5 per group) primed with Pfs48/45 and Pvs48/45 in CFA, respectively were boosted 3 weeks later with the homologous proteins in IFA as the control groups (Pfs48/45 prime–Pfs48/45 boost or Pvs48/45 prime–Pvs48/45 boost). Blood was collected on day 0 and 2 weeks after primary and booster immunizations.

### Analysis of antibody titers, isotype and avidity by ELISA

The antibody titers in the mouse sera were determined by standardized enzyme-linked immunosorbent assay (ELISA) [13] using horseradish peroxidase (HRP)-labeled anti-mouse IgG (GE Healthcare). The endpoint titers were calculated to be the highest serum dilution that gave an absorbance value greater than the mean plus 3 standard deviations (SD) of the absorbance values of pooled pre-immune sera in each assay. In preliminary studies, we had also used pooled sera from mice immunized with adjuvant alone and the absorbance values were similar to those with pre-immune sera. Peroxidase conjugated anti-mouse IgG1, IgG2a, IgG2b and IgG3 (Southern Biotech, 1:2500 dilution) were used as secondary antibodies for antibody isotype analysis. For comparing relative avidity of antigen-antibody interaction, ELISA plates after incubation with primary antibodies were treated with NaSCN (0, 0.5, 1.0, 2.0, 4.0, 8.0 M) for 15 minutes prior to incubation with the secondary antibody (HRP-labeled anti-mouse IgG). Binding of antibodies after NaSCN treatment was expressed as percent of total binding (without NaSCN) and NaSCN concentration resulting in 50% dissociation was used as avidity index.

### Western blot analysis

Purified recombinant Pfs48/45 and Pvs48/45 were fractionated by 12.5% SDS-PAGE, whereas 15% SDS-PAGE was used to fractionate five sub-fragments of Pfs48/45. The proteins were transferred to nitrocellulose membranes and blocked with 5% non-fat milk in PBS containing 0.05% Tween-20 (PBST, pH 7.4). Membranes and strips were probed with various antisera at indicated dilutions and developed using HRP-conjugated anti-mouse IgG antibody (1:10,000 dilution) and ECL Prime Western blotting detection reagent (GE Healthcare).

*P*. *falciparum* (NF54) gametocytes were purified (>90% enriched) using Miltenyi LD paramagnetic column. Purified gametocytes were lysed in SDS-PAGE sample buffer (non-reducing) and fractionated using Bio-Rad mini-Protean TGX precast gels. Proteins were transferred to nitrocellulose membranes and probed as above.

### Indirect immunofluorescence assays (IIFA)

Gametocytes of *P*. *falciparum* (NF54) were harvested from *in vitro* culture, and gametes were purified after incubating gametocytes in the exflagellation buffer followed by purification using discontinuous Nycodenz gradient [3]. Purified parasites were then spotted on multi-well slides (Carlson Scientific) and stored at -80°C. For IIFA, slides were fixed with acetone at -20°C for 20 minutes, blocked with 1% non-fat milk in PBS for 20 minutes, followed by incubation with various sera at 1:100, 1:500 and 1:1,000 dilutions for 30 minutes at room temperature. Sera from pre-immune mice or from mice immunized with adjuvant alone were used as negative controls. FITC-conjugated anti-mouse IgG antibody (Sigma) was used at 1:100 dilution (30 minutes at room temperature) to detect binding of antibodies to parasites. Parasite nuclei were stained by NucBlue Fixed Cell ReadyProbes Reagent (Life technology) as per product manual. The slides were examined using a fluorescence microscope (Olympus BX41) at 1000X magnification (100X oil immersion) equipped with QIClic CCD Camera (QImaging) and QCapture Pro 7 Software (QImaging).

Gametes of *P*. *falciparum* used in live IFA [3, 13] were purified as above by Nycodenz gradient centrifugation. Purified live gametes were incubated with 1:100 dilution (RPMI medium containing 1% non-fat milk) of various mouse sera (immune and pre-immune) with intermittently gentle mixing for 45 minutes on ice. Cells were washed three times with chilled RPMI (2 min at 3,000 rpm, ‘Immufuge II’, Baxter Inc.) and incubated with 1:100 dilution of FITC-conjugated goat anti-mouse IgG antibody (Southern Biotech) for 45 minutes on ice. NucBlue Fixed Cell ReadyProbes Reagent (Life technology) was used as per product manual and no DAPI staining was seen indicating viability of gametes through purification and staining procedure. After washing, parasites were examined and images were captured and processed as above.

### Ethical statement

All the research on mice adhered to the “Principles of Laboratory Animal Care” (NIH publication #85–23, revised in 1985) and were reviewed and approved by the Institutional Animal Care and Use Committee of Tulane University with an approved protocol number 4172R. Animals were monitored weekly for any adverse effects and none of the animal became ill or died. At the end of the study period, animals were anesthetized with isoflurane for terminal blood collection by cardiac puncture.

## Results

### Cross-reactivity between Pvs48/45 and Pfs48/45 using immune mouse sera

**Fig 1:**
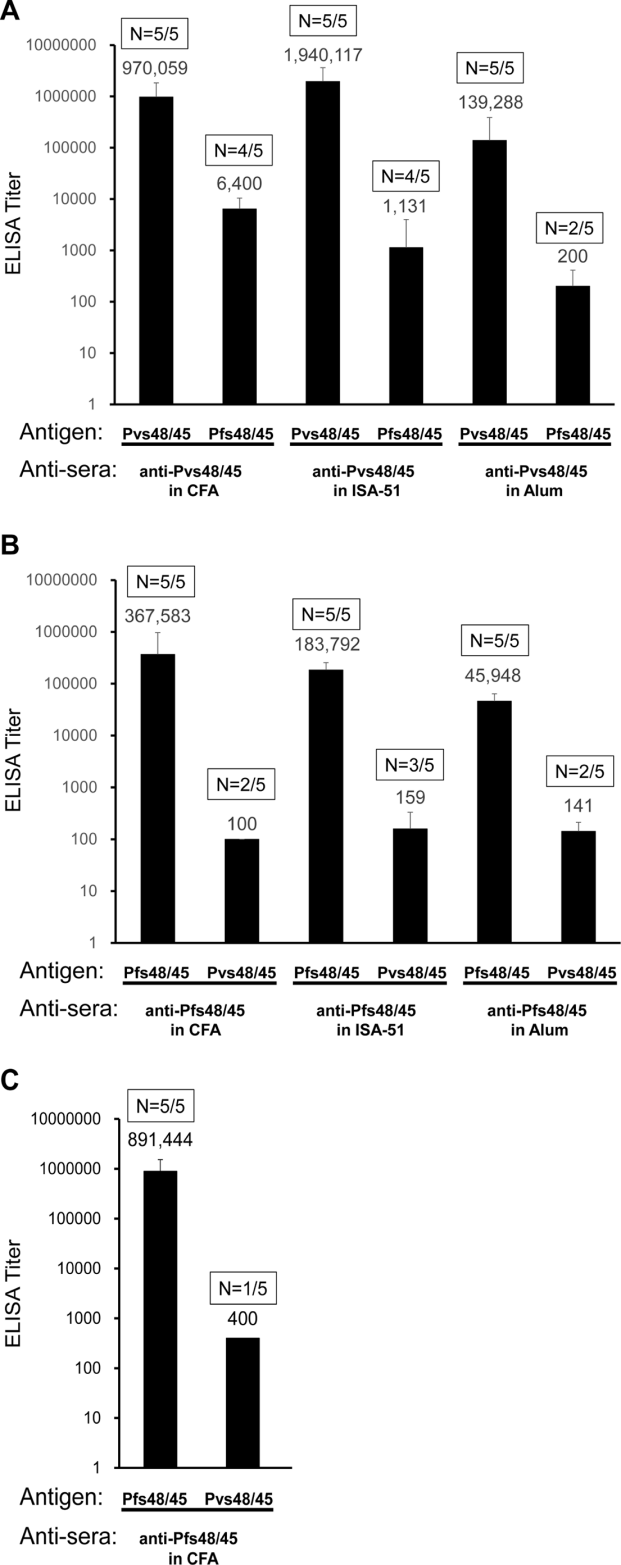
Cross-reactivity of Pvs48/45 and Pfs48/45 immune mouse sera in ELISA. (A) Anti-Pvs48/45 sera from BALB/c mice after 3-dose protein immunization in three adjuvants (CFA, Montanide ISA-51 and Alum) were tested for reactivity against Pvs48/45 and Pfs48/45 antigens by ELISA. (B) Anti-Pfs48/45 sera from BALB/c mice after 3-dose protein immunization in three adjuvants were tested for reactivity against Pfs48/45 and Pvs48/45 antigens by ELISA. (C) ELISA results using anti-Pfs48/45 sera from C57BL/6 mice after 3-dose protein immunization in CFA tested for reactivity against Pfs48/45 and Pvs48/45 antigens. Antigens used for ELISA analysis are identified below each column. The geometric mean ELISA titers are shown above each column. The error bars indicate SD. The numbers within rectangles above each column show number of positive mouse sera / total mice tested.

The P48/45 proteins share about 55% identity of amino acid sequence between *P*. *falciparum* and *P*. *vivax* (S1 Fig). However, immune cross-reactivity of P48/45 from *Plasmodium* species has not been reported before. To confirm the possibility of immune cross-reactivity, the immune sera from mice immunized three times (primary and two booster doses) with Pfs48/45 or Pvs48/45 recombinant antigens using three different adjuvants (CFA, Montanide ISA-51 and Alum) were employed in the ELISA analysis to determine antibody titers against both antigens. All the mice after immunization with Pvs48/45 and Pfs48/45 responded with higher ELISA titers against Pvs48/45 and Pfs48/45, respectively (Fig 1, panels A and B). When tested for cross-reactivity against Pfs48/45, sera from mice immunized with Pvs48/45 using CFA showed the highest geometric mean antibody titer against Pfs48/45 (6400 for 4/5 mice), followed by sera from Montanide ISA-51 (1131 for 4/5 mice) and Alum (200 for 2/5 mice) (Fig 1, panel A). In contrast, when the reverse was investigated, 7 out of 15 mice immunized with Pfs48/45 in three different adjuvants (5 mice each group) revealed very weak (less than 200 ELISA titers) antibody responses against Pvs48/45 (Fig 1, panel B). We also tested sera from C57BL/6 mice that were immunized with Pfs48/45 using CFA and once again only 1 in 5 sera showed weak ELISA positivity (end point titer of 400) to the Pvs48/45 antigen. These results demonstrated higher immune cross-reactivity of anti-Pvs48/45 sera with Pfs48/45 protein.

**Fig 2:**
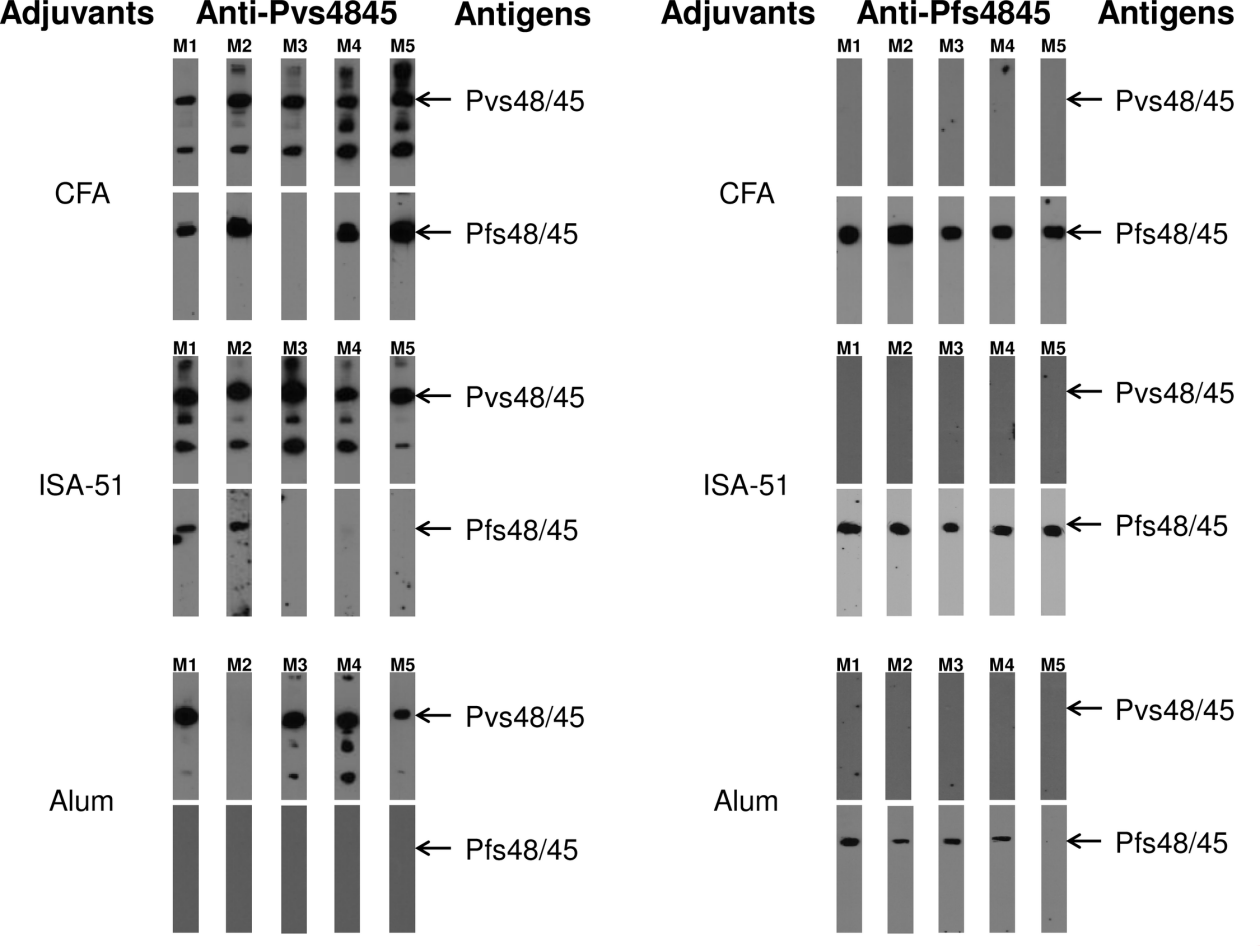
Western blotting analysis of mouse anti-sera with the full-length Pfs48/45 and Pvs48/45 recombinant antigens. Left panels show Western blotting results with anti-Pvs48/45 sera from BALB/c mice (N = 5 per group, M1 –M5) immunized respectively with 3 adjuvants (CFA, Montanide ISA-51 and Alum). Each serum was tested at 1:1,000 dilution for recognition of Pfs48/45 antigen and at 1:10,000 dilution for recognition of Pvs48/45 antigen. The right panels show Western blotting results with anti-Pfs48/45 sera from BALB/c mice (M1 –M5) immunized using CFA, Montanide ISA-51 and Alum. The dilution of each serum was 1:1,000 for recognition of Pvs48/45 antigen, and 1:10,000 for recognition of Pfs48/45 antigen.

In order to confirm cross reactivity patterns observed by ELISA we further tested immune-reactivity patterns by Western blotting analysis (Fig 2). The purified recombinant Pfs48/45 and Pvs48/45 proteins were fractionated by SDS-PAGE and reacted with mouse antisera. All four ELISA positive anti-Pvs48/45 sera from mice immunized in CFA group showed strong recognition of Pfs48/45 and the mouse serum that was negative in ELISA did not react. Next we analyzed immune sera from mice immunized with Pvs48/45 formulated in Montanide ISA-51 and Alum adjuvants to detect the cross-reactivity to Pfs48/45 antigen. Only two out of five anti-Pvs48/45 sera from Montanide ISA-51 adjuvant group showed recognition of Pfs48/45 antigen and none of the five antisera from Alum adjuvant group recognized Pfs48/45. Later in these studies we present further evidence that the observed cross-reactivity was also detected with native Pfs48/45 in *P*. *falciparum* gametes and gametocytes, and it was not due to common 6X histidine tag present on both recombinant Pfs48/45 and Pvs48/45. These results suggest that Pvs48/45 antigen was able to induce antibodies recognizing cross-reactive epitopes in orthologous antigen of *P*. *falciparum*. However, this cross-reactive immunogenicity is greatly influenced by adjuvants used for immunization as suggested by the induction of quantitatively and qualitatively strongest cross-reactive antibodies with CFA followed by Montanide ISA-51 and none with Alum. A similar Western blotting analysis once again confirmed lack of recognition of Pvs48/45 by all anti-Pfs48/45 sera from mice immunized using three different adjuvants (Fig 2, right panels). We also tested the single serum from C57BL/6 mice that had shown very low ELISA titer (1:400, see Fig 1, panel C), even at the lowest 1:100 serum dilution, and no recognition of Pvs48/45 by Western blotting was observed (data not shown), ruling out the possibility that cross-reactivity was affected by the species of immunized host.

In addition to the recognition of major Pvs48/45 band by anti-Pvs48/45 antisera, Western blotting studies revealed recognition of higher and lower molecular weight bands. Both Pfs48/45 and Pvs48/45 contain 15 to 16 cysteine residues and the recombinant expression products usually form higher order aggregates when analyzed under non-reducing SDS-PAGE conditions. However, all these molecular forms converge into a single major species under reducing SDS-PAGE (S2 Fig, panel A). These are all specific protein bands as revealed by WB results using anti-His antibodies (S2 Fig, panels B and C). Intriguingly, Pvs48/45 during expression in *E*.*coli* appears to undergo partial proteolytic breakdown giving rise to the presence of a smaller protein product of approximately 24–30 kDa. This band can be seen: (i) under reducing SDS-PAGE conditions, (ii) in C-terminal His tagged Pvs48/45 (S2 Fig, panel A) used for all the studies here as well as when His tag was inserted towards the N-terminus (S2 Fig, panel D) (unpublished), and (iii) is recognized by anti-His antibody (S2 Fig, panels C and E). A similar size band was also found to be produced after limited tryptic digestion of Pfs48/45 protein in gametocyte extract [27]. These results provide the explanation for additional bands seen in some of the WB lanes and they are not bacterial contaminating proteins.

### Comparison of isotypes and avidity of antibodies in anti-Pvs48/45 sera recognizing Pvs48/45 and Pfs48/45 antigens

**Fig 3:**
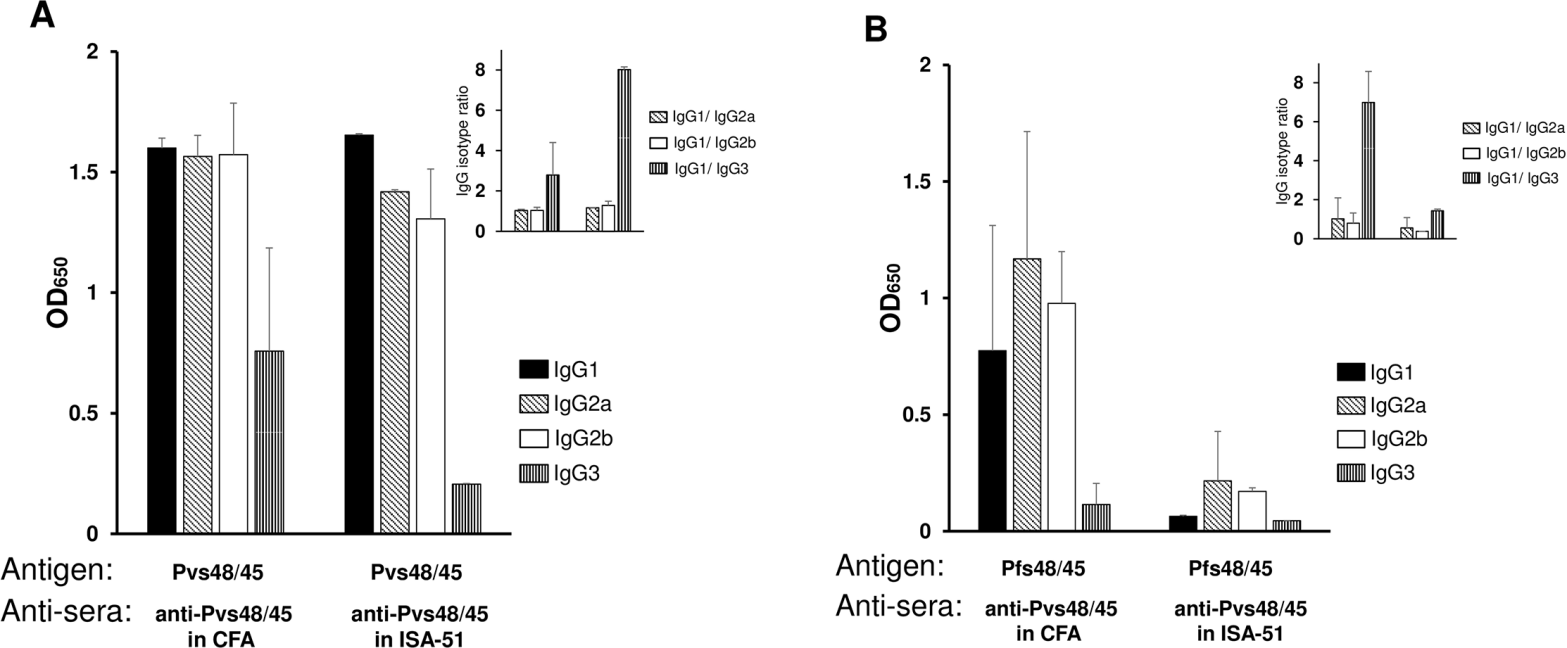
Analysis of antibody isotypes. Anti-Pvs48/45 sera showing positive reactivity to Pfs48/45 in ELISA and Western blotting (4 out of 5 from CFA group and 2 out of 5 from Montanide ISA-51 group) were individually tested to compare immunoglobulin isotypes. ELISA plates coated with Pvs48/45 (panel A) or Pfs48/45 (panel B) were incubated with sera (1:10,000 dilution for Fig 3A and 1:100 dilution for Fig 3B). The plates were then incubated with peroxidase-conjugated goat anti-mouse IgG1, IgG2a, IgG2b and IgG3 (1:2,500 dilution) and processed as in standard ELISA. Shown are mean absorbance values for each isotype and the insets in panels A and B show relative proportions of IgG2a, IgG2b and IgG3 isotypes compared to IgG1. The error bars indicate SD.

In view of the observed cross reactivity of anti-Pvs48/45 sera with Pfs48/45, we further analyzed anti-Pvs48/45 sera to compare isotypes and avidity of antibodies. Isotype analysis revealed equivalent predominance of IgG1, IgG2a and IgG2b isotypes of antibodies recognizing either homologous (Pvs48/45) or heterologous (Pfs48/45) antigens with IgG1/IgG2a or IgG1/IgG2b ratios around 1.0. Our analysis also revealed the high presence of IgG3 isotype against Pvs48/45 in anti-Pvs48/45 sera, however the same was much diminished when anti-Pvs48/45 sera were analyzed against Pfs48/45 (Fig 3A and 3B).

**Fig 4:**
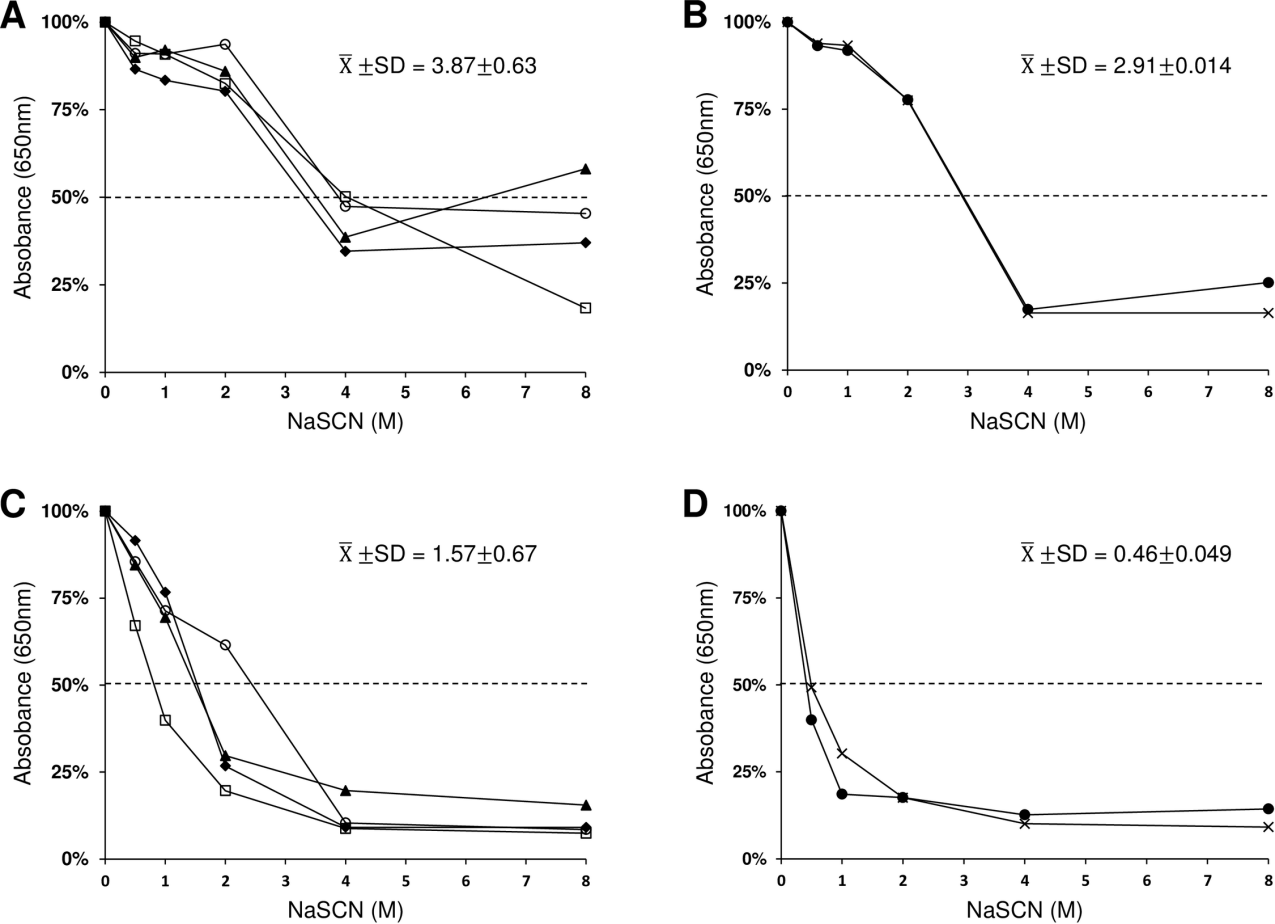
Analysis of avidity. Anti-Pvs48/45 sera showing positive reactivity to Pfs48/45 in ELISA and Western blotting (4 out of 5 from CFA group and 2 out of 5 from Montanide ISA-51 group) were individually tested to compare avidity of antibodies. ELISA plates coated with Pvs48/45 or Pfs48/45 were incubated with sera at 1:10,000 dilution for Pvs48/45 and at 1:100 dilution for Pfs48/45. Absorbance values after NaSCN treatment were converted to percent of total binding (absorbance without NaSCN) and avidity index was deduced from the molar concentration of NaSCN resulting in 50% dissociation of bound antibody. (A) 4 sera in CFA group against Pvs48/45. (B) 2 sera in ISA-51group against Pvs48/45. (C) 4 sera in CFA group against Pfs48/45. (D) 2 sera in CFA group against Pfs48/45. The mean and standard deviation $\left(\bar{\text{X}}\pm \text{SD}\right)$ of avidity index are shown in each panel. Statistical significance was determined by Student’s t-test with p values <0.05.

NaSCN mediated dissociation of antigen-antibody complex was used to compare avidity of antibodies recognizing Pvs48/45 and Pfs48/45. The avidity index, expressed as NaSCN concentration resulting in 50% dissociation of bound antibody was 2.5 times higher for antibodies bound to homologous Pvs48/45 antigen as compared to Pfs48/45 in CFA group (P = 0.0025) (Fig 4A and 4C), and 6.3 times higher in Montanide ISA-51 group (P = 0.00022) (Fig 4B and 4D), suggesting lower binding avidity of cross reactive antibodies in the sera from Pvs48/45 immunized mice.

### Localization of immuno-reactive Pfs48/45 domains recognized by anti-Pvs48/45 antisera

**Fig 5:**
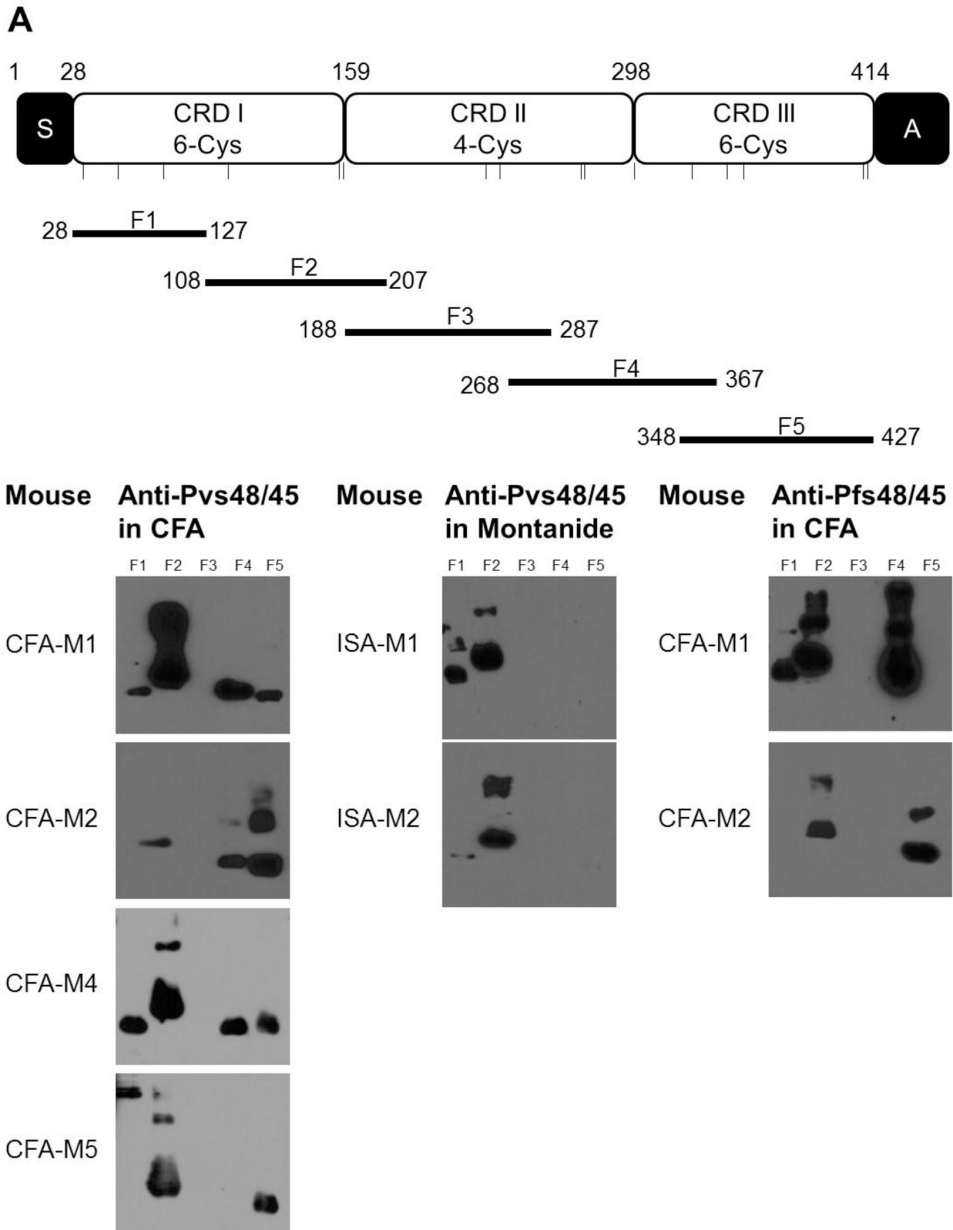
Reactivity of anti-Pvs48/45 sera to recombinant sub-fragments of Pfs48/45. (A) Schematic representation of Pfs48/45 and the amino acid boundaries of five sub-fragments (F1, F2, F3, F4 and F5) of Pfs48/45. S, secretory signal sequence; A, anchor sequence; CRD, cysteine-rich domain. Bars show the relative positions of cysteine residues. (B) Western blotting analysis of the six anti-Pvs48/45 sera from CFA (CFA-M1, CFA-M2, CFA-M4 and CFA-M5) and Montanide ISA-51 (ISA-M1, ISA-M2) adjuvant groups with five overlapping fragments of Pfs48/45. All the anti-Pvs48/45 sera were tested at 1:1,000 dilution. The two anti-Pfs48/45 sera (CFA-M1, CFA-M2) were employed as positive control at a dilution of 1:10,000.

Next, we wished to investigate cross-reactivity of antibodies induced by Pvs48/45 protein at sub-domain level in Pfs48/45. We expressed five sub-fragments (F1-F5) (Fig 5A) of Pfs48/45 in *E*. *coli* and tested by Western blotting using anti-Pvs48/45 sera which had shown strong recognition (ELISA and Western blot) of full length Pfs48/45. The amino-terminal and carboxyl-terminal fragments were designed without signal and anchor sequences, respectively. As shown in Fig 5B, the cross-reactivity was not confined to any dominant region: all four anti-Pvs48/45 sera from CFA adjuvant group recognized Pfs48/45 fragments (F1, F2, F4 and F5) and none of the sera recognized fragment F3. A similar reactivity pattern was also revealed by two anti-Pfs48/45 sera that were used as positive control, thus demonstrating a comparable pattern of immuno-reactive epitopes in Pfs48/45 recognized by homologous (anti-Pfs48/45) and heterologous (anti-Pvs48/45) antisera. Similar studies with the two anti-Pvs48/45 sera from Montanide ISA-51 adjuvant group also revealed recognition of fragments F1 and F2 of Pfs48/45. Further analysis of reactivity pattern suggested that Pfs48/45 fragment F2, recognized by all six cross-reactive anti-Pvs48/45 sera, probably contains major cross-reactive epitopes regardless of adjuvant used for immunization (Fig 5B).

### Confirmation of cross-reactivity by IIFA on the surface of parasites

**Fig 6:**
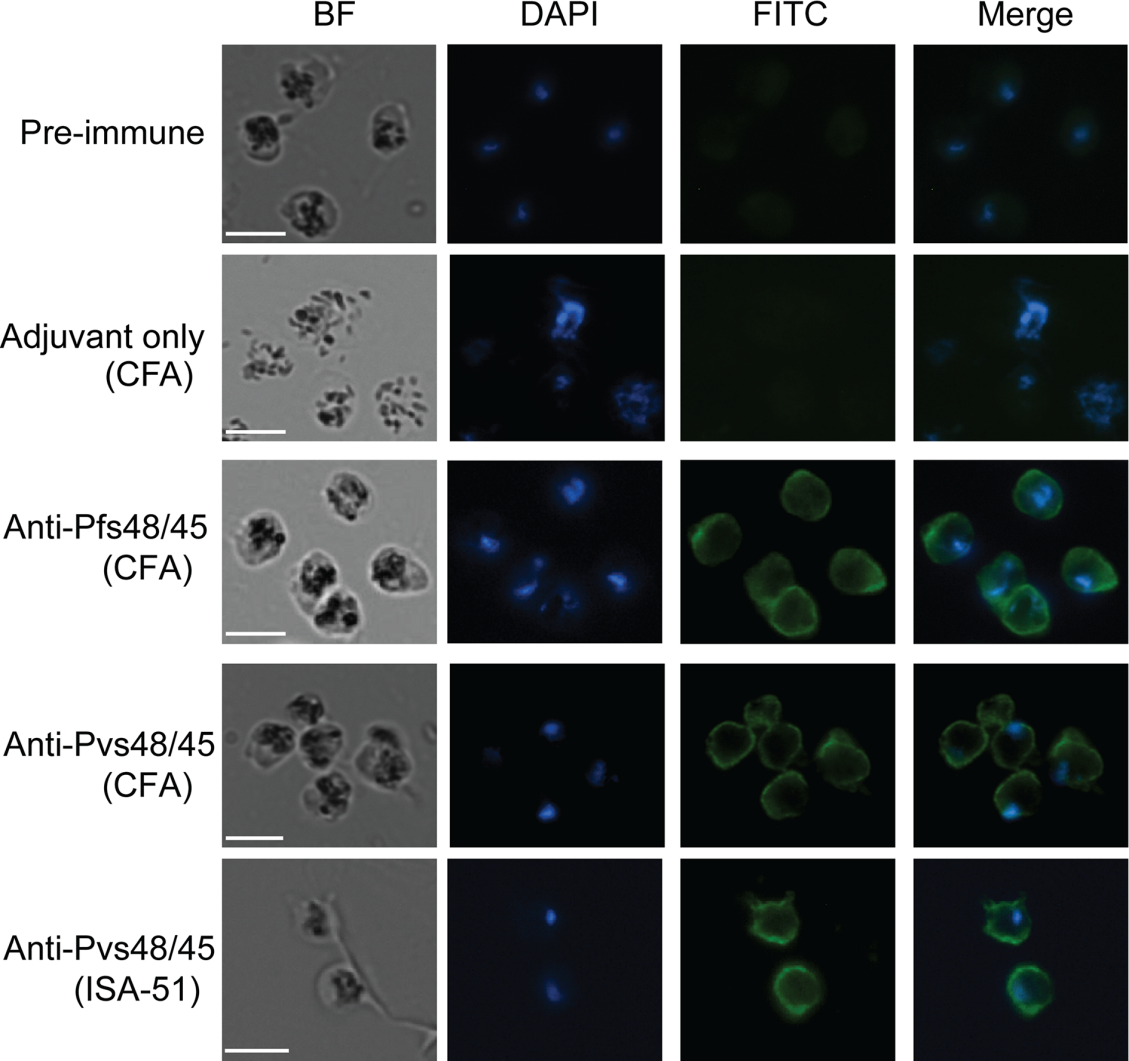
Recognition of Pfs48/45 in fixed *P*. *falciparum* parasites in indirect immunofluorescence assays. The representative results for fixed parasites incubated with pooled pre-immune sera, pooled sera from CFA adjuvant alone immunized mice, a representative anti-Pfs48/45 serum, a representative ELISA and WB positive anti-Pvs48/45 serum (CFA group), and a representative ELISA and WB positive anti-Pvs48/45 (Montanide ISA-51 group) at a dilution of 1:100. BF, bright field; DAPI, the nuclei stained by NucBlue reagent; FITC, antibody reactivity to the parasite visualized with FITC-conjugated anti-mouse IgG antibody, and Merge of DAPI and FITC images. All images were visualized and captured at 1000X magnification. Scale bar (white line), 5μm.

Previous ELISA and WB studies demonstrated immunological cross-reactivity between P48/45 proteins of *P*. *vivax* and *P*. *falciparum*. Next we wished to establish the same at the level of recognition of antigen expressed in the sexual stages of *P*. *falciparum* parasites. We investigated reactivity of anti-Pvs48/45 sera by: (i) IIFA using fixed gametes/ gametocytes, (ii) IIFA on live gamete surface, and (iii) recognition of Pfs48/45 under non-reducing Western blotting conditions in the gametocytes. Two different sera from mice immunized with Pvs48/45 in CFA group and Montanide ISA-51 group were tested, initially at three different dilutions (1:100, 1:500 and 1:1000). Anti-Pfs48/45 and anti-Pvs48/45 sera at 1:100 dilution showed strong reactivity on the surface of fixed *P*. *falciparum* parasites, whereas the pooled pre-immune sera or pooled sera from mice immunized with CFA adjuvant alone did not show any specific reactivity. Dose dependence of recognition was demonstrated by declining fluorescence intensity at higher sera dilutions (not shown). Anti-Pfs48/45 sera in CFA group used as a positive control, as expected, showed the strongest reactivity. These qualitative IIFA results further established that the observed cross reactivity was indeed directed against epitopes exposed on the surface of parasites (Fig 6).

**Fig 7:**
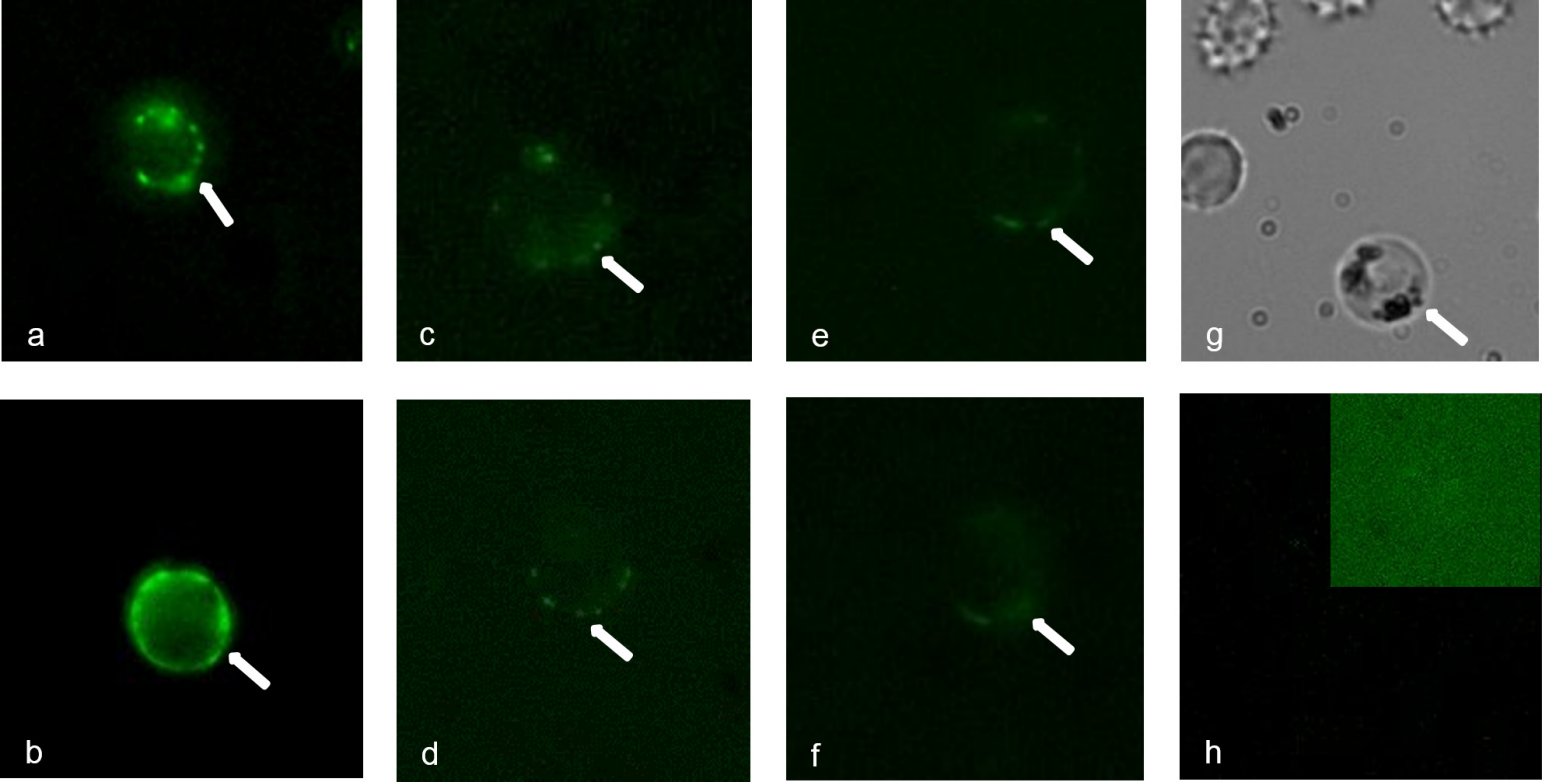
Recognition of Pfs48/45 by anti-Pvs48/45 antisera on the surface of live *P*. *falciparum* gametes. Individual mouse sera were tested (1:100 dilution) by live IFA. Panels a-b show reactivity patterns obtained with two different anti-Pfs48/45 sera. Panels c-f show patterns of positive reactivity of anti-Pvs48/45 sera in live IFA. Panel g shows a representative bright field image of live gamete preparation used and panel h shows the result with a pool of pre-immune mouse sera also tested at 1:100 dilution. The inset in panel h is included to show lack of any detectable reactivity even after altering brightness and contrast settings.

**Fig 8:**
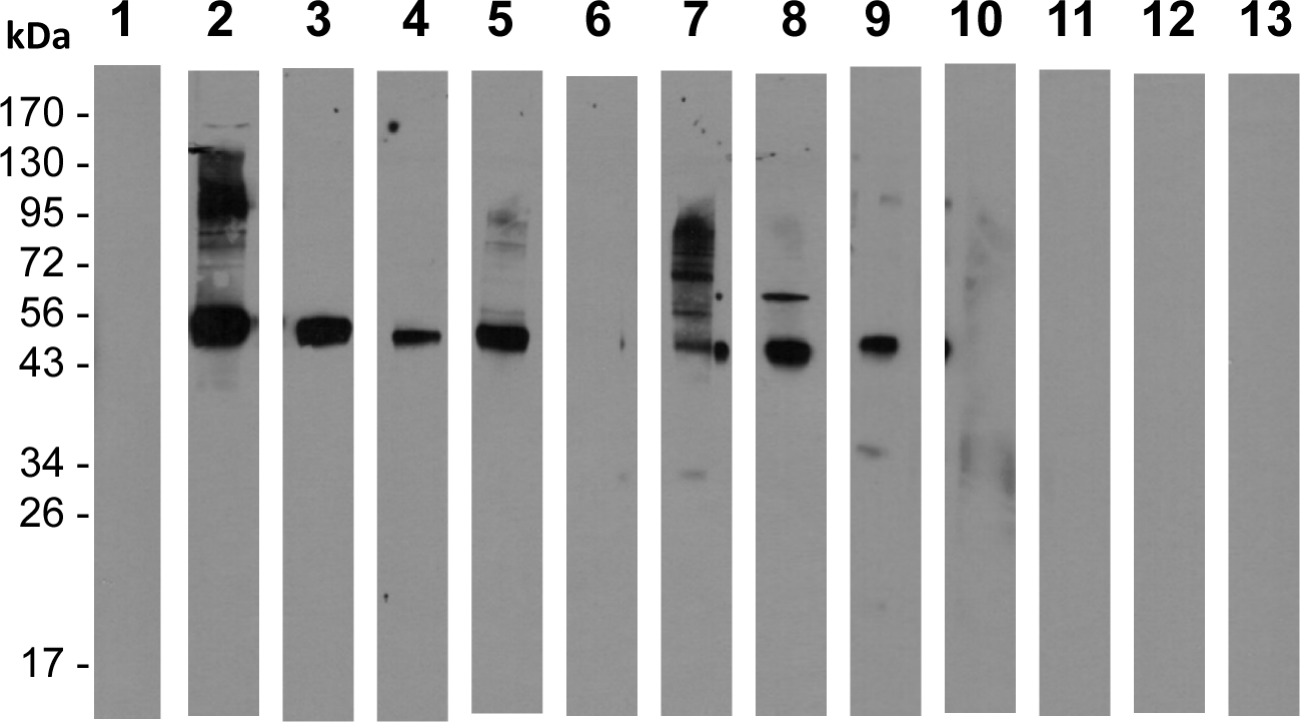
Recognition of Pfs48/45 by anti-Pvs48/45 antisera under non-reducing conditions by Western blotting using purified gametocytes of *P*. *falciparum*. Approximately 1 million gametocytes (*P*. *falciparum*, NF54) per lane were used under non-reducing condition of SDS-PAGE for Western blotting. Lane 1 shows results with a pool of pre-immune mouse sera (1:1000 dilution). Lanes 2 and 3 show results with two different anti-Pfs48/45 sera tested at 1:5000 dilution. Immune sera from five mice (M1-M5 in Figs 1 and 2) immunized with Pvs48/45 using CFA as adjuvant were evaluated at 1:1000 dilution (lanes 4–8). The serum from M3 in this CFA group, which did not display any cross-reactivity in Western blotting and ELISA, also did not detect Pfs4845 antigen in the gametocytes (lane 6). Lanes 9–13 show results on the lack of recognition of Pfs47 by four (M1, M2, M3 and M4) out of these five anti-Pvs48/45 sera tested (1:1000 dilution) using reduced form of *E*. *coli* expressed Pfs47 protein. Pooled sera from mice immunized with rPfs47 using CFA as an adjuvant was used as a positive control (lane 9). Recombinant Pfs47 (unpublished) used was expressed in *E*.*coli* as 6X His tagged protein using pET (K-) expression vector and purified exactly as Pfs48/45 and Pvs48/45 proteins used in these studies.

To further establish cross-reactivity on the surface of live parasites, we also tested anti-Pvs484/45 sera using live gametes of *P*. *falciparum* in IIFA. Purified live gametes showed definite positive surface reactivity and as expected the fluorescence intensity was weaker as compared to the intensity with homologous (anti-Pfs48/45) anti-sera (Fig 7). The pattern of reactivity ranged from continuous surface reactivity to punctate pattern of reactivity on the surface. Finally, anti-Pvs48/45 sera showed strong recognition of Pfs48/45 present in the gametocyte extract and the same sera failed to recognize (Fig 8) recombinant Pfs47, a related molecule belonging to six-cysteine protein family, expressed as His-tagged recombinant protein in *E*. *coli*. Taken together, these results provide further evidence for cross-reactivity of anti-Pvs48/45 sera with Pfs48/45 and that this cross-reactivity exists even at the level of recognition of epitopes exposed on the surface of live gametes.

### Cross boosting of immune responses primed by Pfs48/45 and Pvs48/45

**Fig 9:**
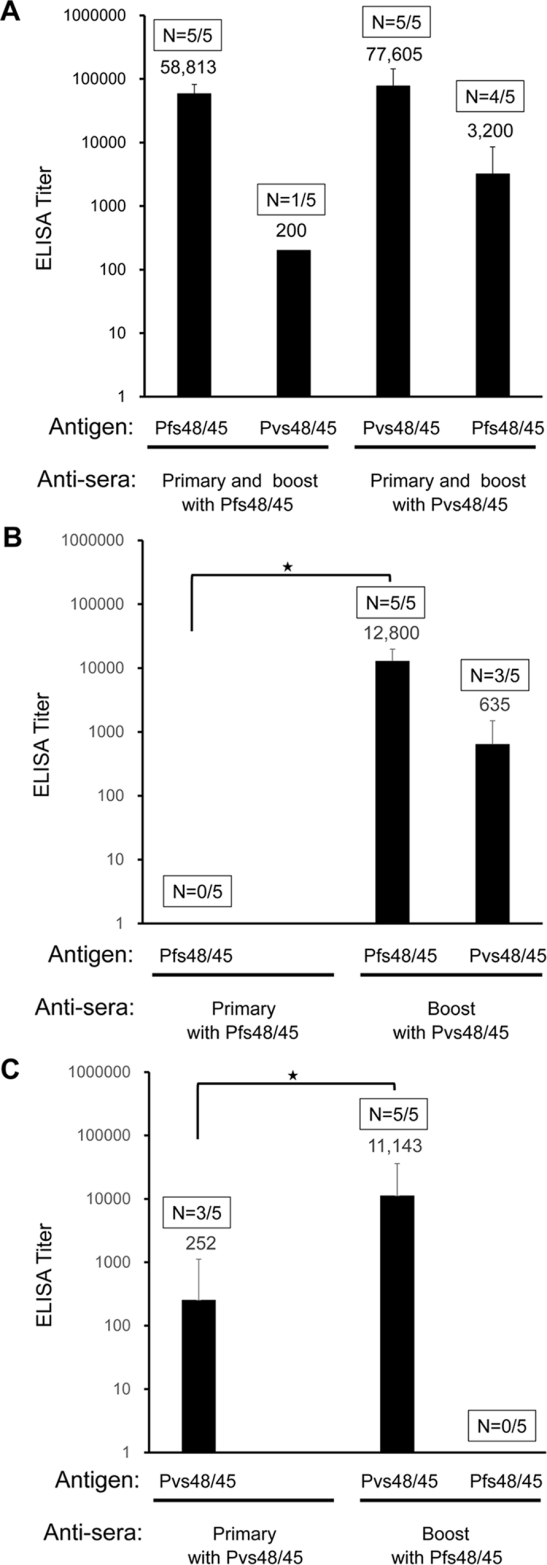
Cross boosting of Pfs48/45 and Pvs48/45 antibody responses. (A) Groups of mice were primed and boosted with the same antigen, either Pfs48/45 or Pvs48/45. Antibody titers were determined against both antigens by ELISA. The data shown are from mice after the booster immunization. (B) Shows results of mice primed with Pfs48/45 and boosted with Pvs48/45. Antibody titers were determined both after the primary and booster immunizations using ELISA plates coated with either Pfs48/45 or Pvs48/45. Antibody titers against Pfs48/45 between prime and boost were statistically significant (p = 0.0055). As shown, Pfs48/45 specific antibody titers after priming with Pfs48/45 were ≤ 100, we used a value of 100 for statistical comparison with titers after boost with Pvs48/45. (C) Shows results of mice primed with Pvs48/45 and boosted with Pfs48/45. Antibody titers were determined both after the primary and booster immunizations using plates coated with either antigen. Antibody titers against Pvs48/45 between prime and boost were statistically significant (p = 0.036)_._ The geometric mean ELISA titer of each group is shown. Boxes above each column show number of positive mice / total mice tested. Statistical significance of the antibody titers between prime and boost were determined by Mann Whitney test with p value < 0.05, indicated by an asterisk (*).

Previous analysis clearly demonstrated cross reactivity of Pvs48/45-induced antibodies with epitopes on Pfs48/45, thus raising the possibility that immune responses primed by Pfs48/45 could be boosted by Pvs48/45 and *vice versa*, i.e. cross-boosting. To test whether Pfs48/45 and Pvs48/45 can boost the immune response primed by Pvs48/45 and Pfs48/45, respectively, groups of mice were primed with Pfs48/45 or Pvs48/45 and boosted once either with homologous antigens or with heterologous antigens. Mice sera after each immunization were tested by ELISA to determine antibody end-point titers against each antigen. In homologous prime boost groups (Pfs48/45 prime—Pfs48/45 boost or Pvs48/45 prime—Pvs48/45 boost), all the mice, as expected showed strong antibody responses against immunizing homologous antigens. When tested against heterologous antigens, only one out of five anti-Pfs48/45 immunized serum showed very weak cross-reactivity to Pvs48/45 antigen. On the contrary, four out of five anti-Pvs48/45 sera showed strong cross-reactivity to Pfs48/45 antigen (Fig 9A), observations similar to those with sera used in earlier studies shown in Fig 1. In heterologous prime boost groups (Pfs48/45 prime—Pvs48/45 boost or Pvs48/45 prime—Pfs48/45 boost), Pvs48/45 antigen was able to boost the antibody response in the mice primed by Pfs48/45 antigen (Fig 9B), and *vice versa* (Fig 9C), presenting direct evidence for statistically significant cross boosting of antigen-specific immune responses by P48/45 antigens.

## Discussion

Many *Plasmodium* antigens, show partial sequence and functional conservation across species, raising the possibility that the immunity induced by such conserved antigens could result in cross-reactive immune responses or even cross-species protection. Such conservation and possible immunological outcome become even more significant, especially since many such *Plasmodium* antigens are also among the leading vaccine candidates. To date, no cross-reactivity for sexual-stage antigens or vaccine candidates for malaria transmission blocking has been reported in the human and murine *Plasmodium* species. The studies reported in this paper addressed the question of immune cross-reactivity between Pfs48/45 and Pvs48/45 proteins, currently being pursued as malaria transmission-blocking vaccine candidate antigens [1, 4, 13, 15, 27, 28]. These two proteins share greater than 50% sequence identity, thus raising the possibility of immunological cross-reactivity between the two, investigated in this study using specific anti-sera and purified recombinant proteins in the ELISA and Western blotting assays.

In our study, the cross-species immune reactivity between Pfs48/45 and Pvs48/45 was confirmed by the fact that anti-Pvs48/45 sera cross-reacted with recombinant Pfs48/45 in both ELISA and Western blotting, however in ELISA, the cross-reactive antibody titers against Pfs48/45 were much lower than those against homologous antigens. Overall, the dominant isotypes of antibodies in anti-Pvs48/45 sera reacting with Pvs48/45 and Pfs48/45 were comparable, except that the proportion of IgG3 in anti-Pvs48/45 sera cross-reacting with epitopes in Pfs48/45 antibodies was considerably reduced. We also compared the avidity of antibodies in anti-Pvs48/45 sera by NaSCN dissociation method and it was found to be significantly lower for Pfs48/45 as compared to that of antibodies reacting with Pvs48/45. Additionally, anti-Pvs48/45 antibodies showed strong recognition of native protein on the surface of live *P*. *falciparum* gametes in IIFA. In similar analysis, anti-Pfs48/45 failed to demonstrate the reverse, i.e. recognition of Pvs48/45 in both ELISA and by Western blotting, except for one sample that showed weak ELISA titer (less than 400). This is somewhat surprising observation and requires further investigation, especially analyzing immune sera from additional strains of mice and higher mammals. On the other hand, it is also possible that cross-reactive epitopes in Pvs48/45 are more immunogenic when presented to the immune system as compared to those epitopes in the context of Pfs48/45, which in turn might suggest that structural differences lead to differential antigen processing and presentation of epitopes to helper T cells.

Western blotting analysis using overlapping fragments of Pfs48/45 was initially expected to define regions exhibiting cross-reactivity. While fragment F2 was found to be recognized by all six out of ten ELISA positive anti-Pvs48/45 sera, fragment F3 was not recognized at all by any of the sera. Fragment F1, F4 and F5 were also recognized differentially by some of the sera tested. A similar pattern of recognition has been observed in previous observation in which anti-Pfs48/45 immune sera were tested against the same panel of Pfs48/45 fragments [29]. Previous studies have shown that the two cysteine-rich subdomains of Pfs48/45 at the amino- and carboxyl- termini can induce transmission blocking antibody responses in experimental animals [12, 27]. Cross-reactivity at the level of amino-terminal (fragments F1 and F2) and carboxyl-terminal overlapping fragments (fragment F4 and F5) corresponding to two cysteine-rich subdomains, CRDI and CRDIII, indicates rather broadly dispersed cross-reactive epitopes between Pvs48/45 and Pfs48/45 on the one hand, and apparent poor immunogenicity of CRDII which corresponds to fragment F3, on the other hand. Additionally, we have also sought to understand why Pfs48/45 fragment F2 was recognized by most cross-reactive anti-Pvs48/45 antibodies. We compared corresponding amino acid sequences of five fragments between *P*. *falciparum* and *P*. *vivax*, and the percent identity was quite similar- 56.9, 60.2, 56, 52 and 62.8 for fragments F1, F2, F3, F4 and F5, respectively, thus ruling out any sequence based preferential recognition.

Another critical observation was the effect of different adjuvants on the observed cross reactivity. Maximum cross-reactivity was revealed by mice immunized in CFA, followed by Montanide ISA-51 and none by alum. Differences in the antibody titers against Pvs48/45: geometric mean ELISA titer 970059 in CFA, 1940117 in Montanide ISA-51 and 139288 in alum cannot explain the pattern of cross-reactivity seen. The anti-Pvs48/45 sera in alum group did not show any cross-reactivity by Western blotting to heterologous Pfs48/45 antigen. Likewise, anti-Pvs48/45 sera in Montanide ISA-51 group, despite the highest Pvs48/45-specific antibody response in ELISA, revealed: weaker and only half as many mice showing cross-reactivity as compared to the CFA group; and the reactivity was confined to the fragments F1 and F2 in the amino- terminal cysteine-rich subdomain of Pfs48/45. While this observation may be rather preliminary it is tempting to speculate that different adjuvants seem to influence presentation of epitopes in Pvs48/45, resulting in the difference in the recognition of cross-reactive epitopes in Pfs48/45.

In this study, we present evidence for immune cross-reactivity of P48/45 between *P*. *falciparum* and *P*. *vivax*, two species of *Plasmodium* responsible for ~90% of all malaria infections in the world and co-endemic in many countries, especially in S.E. Asia and Central and S. America [26]. Since the levels of cross-reactive antibodies in anti-Pvs48/45 sera revealed much lower ELISA titers against Pfs48/45, we are not sure if they will be sufficient to cause any functional effect in terms of transmission reduction. However, with repeat immune boosting they might potentially reach blocking levels needed, and as such future studies are needed to address such a possibility. Nonetheless, our studies did indicate that antibodies induced by Pvs48/45 recognize epitopes on the surface of live *P*. *falciparum* gametes, further supporting the notion of possible biological effects. It is well known that targets of transmission blocking antibodies are conformational in nature and further characterization of cross-reactivity needs to investigate such conformational determinants. In this regards, studies are in progress in our lab to develop monoclonal antibodies to characterize the nature of cross-reactive epitopes and to evaluate functional significance of antibodies against any such epitopes. Our results support additional studies to determine if cross-reacting antibodies are also elicited in people naturally infected with either or both *P*. *falciparum* and *P*. *vivax* and whether the cross-reactivity of P48/45 could lead to cross-species transmission blocking activity. Apart from cross reactivity, further biological significance presented in this study comes from the fact that Pfs48/45 and Pvs48/45 were able to cross-boost immune responses against each other. There is thus the possibility that similar boosting can occur by natural infection after priming dose coming in the form of vaccines based on either antigen, and such cross boosting could have a positive impact in maintaining higher antibody titers.

## Supporting Information

S1 Fig Comparison of amino acid sequences of Pfs48/45 and Pvs48/45 proteins. The amino acid sequences were aligned using EMBO Stretcher, the online software provided by EMBL (http://www.ebi.ac.uk/Tools/psa/emboss_stretcher/). Pfs48/45 and Pvs48/45 share 55% identity and 75% similarity in the protein sequences. Conserved cysteine residues are identified by (+) signs, identical amino acid residues by (**|)**, and conserved amino acid residues by (**:).**
(TIF)

S2 Fig SDS-PAGE and Western blot analysis of recombinant Pvs48/45 antigens with the C- and N- terminal (His)_6_ tag. (A). SDS-PAGE analysis of purified Pvs48/45 antigen with C-terminal (His)_6_ tag under non-reduced (NR) and reduced (R) SDS-PAGE conditions. (B). Western blotting of non-reduced Pvs48/45 containing C-terminal (His)_6_ tag using anti(His)_6_- monoclonal antibody. (C). Western blotting of reduced Pvs48/45 containing C-terminal (His)_6_ tag using anti(His)_6_- monoclonal antibody. (D). SDS-PAGE analysis of purified Pvs48/45 antigen containing N-terminal (His)_6_ tag under reduced SDS-PAGE condition. (E). Western blotting of reduced Pvs48/45 containing N-terminal (His)_6_ tag using anti(His)_6_- monoclonal antibody.
(TIF)
