# Supplementary figures and images for: Immunological Cross-Reactivity between Malaria Vaccine Target Antigen P48/45 in Plasmodium vivax and P. falciparum and Cross–Boosting of Immune Responses
Source: PLoS One. 2016 Jul 20;11(7):e0158212. doi: 10.1371/journal.pone.0158212 (PMC4954667; doi:10.1371/journal.pone.0158212)

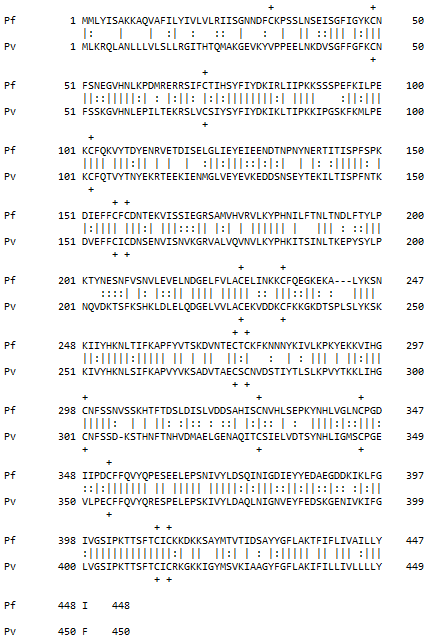

Supplement: S1 Fig — The amino acid sequences were aligned using EMBO Stretcher, the online software provided by EMBL (http://www.ebi.ac.uk/Tools/psa/emboss_stretcher/). Pfs48/45 and Pvs48/45 share 55% identity and 75% similarity in the protein sequences. Conserved cysteine residues are identified by (+) signs, identical amino acid residues by (|), and conserved amino acid residues by (:). (TIF) [file pone.0158212.s001.tif]

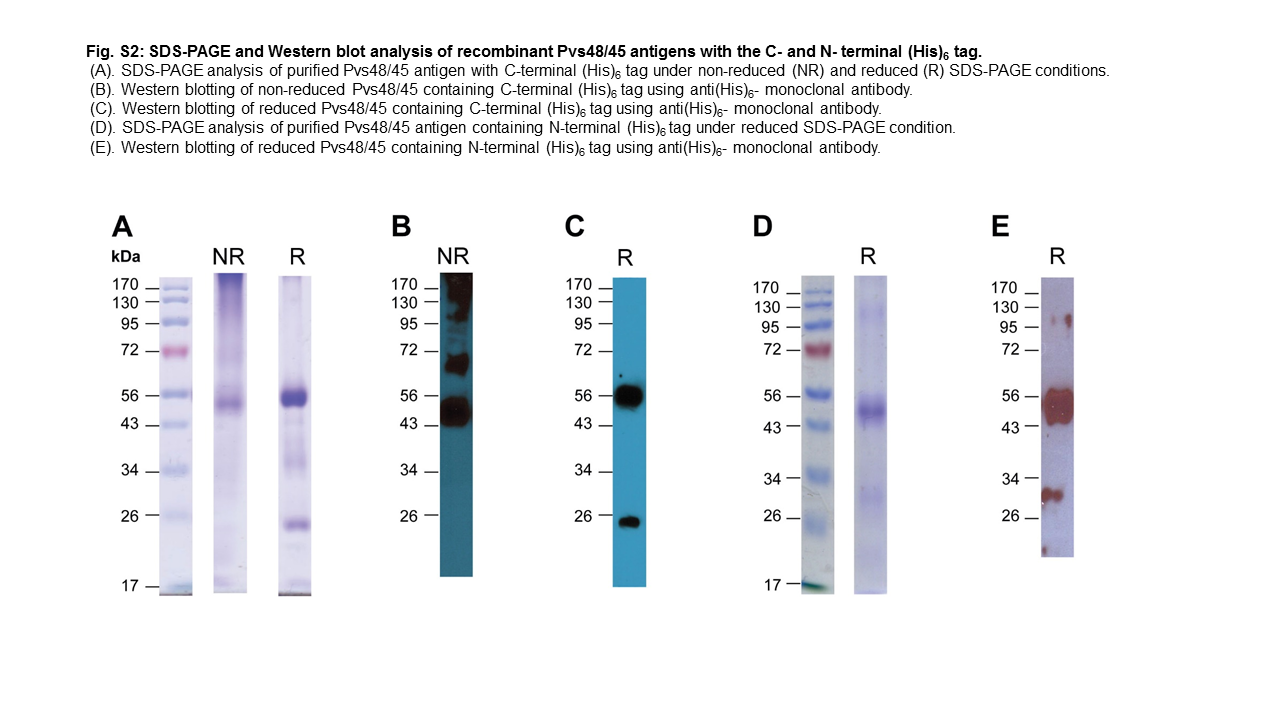

Supplement: S2 Fig — (A). SDS-PAGE analysis of purified Pvs48/45 antigen with C-terminal (His)6 tag under non-reduced (NR) and reduced (R) SDS-PAGE conditions. (B). Western blotting of non-reduced Pvs48/45 containing C-terminal (His)6 tag using anti(His)6- monoclonal antibody. (C). Western blotting of reduced Pvs48/45 containing C-terminal (His)6 tag using anti(His)6- monoclonal antibody. (D). SDS-PAGE analysis of purified Pvs48/45 antigen containing N-terminal (His)6 tag under reduced SDS-PAGE condition. (E). Western blotting of reduced Pvs48/45 containing N-terminal (His)6 tag using anti(His)6- monoclonal antibody. (TIF) [file pone.0158212.s002.tif]
